# Safety and Effectiveness of Vibration Massage by Deep Oscillations: A Prospective Observational Study

**DOI:** 10.1155/2013/679248

**Published:** 2013-10-03

**Authors:** Karin Kraft, Susanne Kanter, Hubert Janik

**Affiliations:** ^1^Chair of Complementary Medicine, Center of Internal Medicine, Rostock University Medicine, Ernst-Heydemann-Straße 6, 18057 Rostock, Germany; ^2^Ernst von Bergmann Clinical Center, Center of Anesthesia, Intensive Care and Surgery Management, Charlottenstraße 72, 14467 Potsdam, Germany

## Abstract

The objective of this study is to assess the safety of treatment with vibration massage using a deep oscillation device and the effects on symptom severity and quality of life in patients with primary fibromyalgia syndrome (FMS). Outpatients with FMS performed an observational prospective study with visits 2–4 weeks after the last treatment (control) and after further 2 months (follow-up). Patients were treated with 10 sessions of 45 min deep oscillation massage, 2/week. Primary outcome parameters were safety and tolerability (5-level Likert scale (1 = very good)) (after each treatment session and at control visit). Secondary outcome parameters were symptom severity (Fibromyalgia Impact Questionnaire (FIQ), pain) and quality of life (SF-36). Seventy patients (97.1% females) were included. At control visit, 41 patients (58.6%) reported 63 mild and short-lasting adverse events, mainly worsening of prevalent symptoms such as pain and fatigue. Tolerability was rated as 1.8 (95% confidence interval: 1.53; 2.07). Symptoms and quality of life were significantly improved at both control and follow-up visits (at least *P* < 0.01). In conclusion, deep oscillation massage is safe and well tolerated in patients with FMS and might improve symptoms and quality of life rather sustained.

## 1. Introduction

Fibromyalgia syndrome (FMS) is a persistent and disabling widespread pain condition accompanied by chronic fatigue, cognitive problems, sleep disturbance, depression, and anxiety [1, 2]. The prevalence of FMS is 0.5–6.6% in North America and Europe, and high health care utilization and costs, especially with pharmacological treatment, have been estimated by various authors [3–6]. Current evidence suggests that centrally augmented pain processing and deficiencies in descending central modulation play an important role in producing pain in FMS; they may also negatively influence sleep, mood, and level of alertness [7]. Although there is no specific peripheral tissue anatomy that characterizes fibromyalgia, this does not reduce the importance of peripheral nociceptive mechanisms [8].

Treatment of FMS is focused on alleviating pain and increasing functioning, as causal therapy is still not available. In a present guideline, a long-term pharmacological treatment with serotonin/norepinephrine reuptake inhibitors or pregabalin is not recommended [2]. Nonpharmacological treatments, in particular lifestyle-oriented intervention including patient education, aerobic or other physical exercise, and cognitive-behavior therapy, have yielded effect sizes and cost-benefit ratios comparable to medications [9–11]. However, a number of challenges prevent the broader adoption of these interventions, such as low compliance and high dropout rates for exercise, loss of benefit at follow-up, if compliance is low, and a lack of availability of these interventions to many patients [11, 12].

Massage therapy is widely used by patients with FMS seeking symptom relief. In a recent narrative review all studies showed short-term benefits of massage, and only one single-arm study demonstrated long-term benefits. All reviewed studies had methodological problems [13]. This resulted in a negative recommendation in a present S3-guideline on FMS [2]. 

In order to alleviate the availability of massage therapy, various technical devices imitating massage techniques are on the market, especially for home-based self-application; however, clinical trials on safety and efficacy in FMS patients are not available for these devices. For example, the effects of manual lymph drainage are claimed to be intensified by deep oscillations of the local tissue, which are generated by moving an applicator with a pulsating electrostatic field [14]. The technique is used by physiotherapists but also promoted for home-based self-application. At present, deep oscillation massage is used to stimulate the absorption of edema, to reduce pain, and to alleviate wound healing as well as for its anti-inflammatory and antifibrous effects [15–17]. Information on safety and tolerability is not available. 

The objective of the present study was the evaluation of safety and tolerability of a series of ten treatments with deep oscillation massage in patients with FMS. For preliminary information on effects, validated scores for measurement of symptoms and functioning and a patient-centered measure were used. 

## 2. Material and Methods

### 2.1. Study Design

An open uncontrolled prospective observational study was performed.

### 2.2. Setting and Patients

Patients were recruited by contacting FMS support groups and by advertisement in local newspapers. They were screened for eligibility by the study physician during a telephone call. Information and the appointment for the baseline visit (I1) were posted to eligible patients. At I1, patients (>18 and <70 years) with primary FMS diagnosed by rheumatologists at least 2 years ago were enrolled; they met the diagnostic criteria of the American College of Rheumatology [18]. Exclusion criteria were Pacemaker or other electronic implants, cardiac failure (stage NYHA III-IV), cancer (except patients free from relapse since 5 years), acute systemic inflammatory diseases, acute and chronic infections (such as HIV, viral hepatitis, and active tuberculosis), fever, acute venous thrombosis, arterial occlusive and other vascular diseases, dermatological diseases (infections, contact allergies, and unclear diagnosis), secondary edema, pregnancy, stillbirth, electromagnetic hypersensitivity, psychoses and current substance abuse, and participation in a clinical study at present or during the last 3 months. From the 93 individuals screened, 70 (68 females, 2 males) met the criteria for inclusion. Informed consent of patients was obtained, fulfilling the ethical criteria established in the Helsinki Declaration, modified in 2000, for research projects. The study was approved by the Institutional Review Board of Ethics at the University of Rostock (reference A 32/2007).

### 2.3. Procedure

At I1, participants were informed in detail and signed the patient information, a consent sheet, and the privacy policy. Demographic baseline data was collected; present and previous therapies of FMS were noted. The Mainz pain staging system (MPSS) was used for the characterization of the stage of chronicity of FMS (range between 1 (low stage) and 3 (high stage)) [19, 20]. Data on endpoints was collected at I1, 2–4 weeks after the last treatment session (I2), and 2 months after I2 (I3, follow-up) by a trained physician and additionally at each treatment session by the treating physiotherapist (Table 1). Visits I1–I3 were scheduled to take place in the morning in order to minimize circadian influences. Patients were instructed at I1 not to reduce any nonpharmacological life-style activities.

### 2.4. Intervention

For deep oscillation massage, a pulsating electrostatic field of low intensity and frequency is created between the manual applicator and the patients' tissue intended to be treated. The rectangular impulse chain controls the attraction of the skin. An active electrostatic impulse enhances the attraction at the off-level the skin is released. Consequently, rhythmic frictions are generated by moving the applicator repeatedly and quickly into the same direction. This results in oscillations of the local tissue including skin, subcutaneous tissue, muscles, blood vessels and lymphatic vessels, and presumably an increased local vascular circulation. In the present study, a portable device (DEEP OSCILLATION Personal, Physiomed, Schnaittach/Laipersdorf, Germany) was used, which is also designed for self-application [21]. Each patient received 10 sessions of deep oscillation massage (45 min/treatment, 2/week) in one of 5 practices of physical therapy by experienced physiotherapists, who previously had been trained on how to handle the device. A standardized treatment (previously fixed frequencies and duration of the application of each frequency) was applied. The manual applicator was moved on the body regions to be treated according to the standard procedure of manual lymph drainage [22]. 

### 2.5. Measures

Safety was evaluated by unwanted event monitoring by the patients. At I1, they were instructed to note all events occurring during or immediately after each treatment session and additionally unfamiliar events from the first treatment until visit I2. The events reported were classified as adverse. Severe adverse events were death, hospitalization, and persisting symptoms, and an unscheduled consultation of a physician immediately after a treatment session because of unexpected reactions.

After each treatment session, patients and therapists rated the tolerability of deep oscillation massage, using a 5-point Likert scale (range from 1 = very good to 5 = very bad); likewise, the tolerability was rated by the patients at I2. Also, patients were asked at I2 and I3 whether they would agree to a further treatment series with deep oscillation massage.

FMS-specific health status was measured with the Fibromyalgia Impact Questionnaire (FIQ) [23]. Its score ranges from 0 to 100 with 3 defined categories of severity: mild (0 to <39), moderate (39 to <59), and severe (59 to 100) [24, 25]. 

Pain related to FMS was assessed at visit I1–3 by a 100 mm visual analogue scale (VAS) [26]. According to Bennett et al., score subdivisions were defined as mild (0 to <40 mm), moderate (40 to 59 mm), severe (60 to 79 mm), and very severe (80 to 100 mm) [25]. Also, pain related to FMS was recorded before and after each treatment session (Table 1). Additionally, patients indicated their individually acceptable pain level by VAS at I1. 

The Short Form-36 Health Status Questionnaire (SF-36) was applied at I1–I3 in order to assess physical and emotional health attributes during the past month [27, 28].

The pain perception scale (SES) is a validated and approved German pain questionnaire. It allows for a differentiated description of chronic pain with respect to its affective and sensory parts and also for recording of changes in the course of time. High score values (affective: 14–56 points, sensory: 10–40 points) reflect high levels of pain sensation. Calculated *T*-scores of the reference sample were used [29]. 

At treatment sessions 1 and 10, the validated Multidimensional Mood State Questionnaire (MDBF) was applied before (version A) and after treatment (version B), which explores 3 bipolar designed dimensions of the current mental condition, each by 4 questions: Good/bad mood (GM), feeling awake/tired (AT), and calm/nervous (CN). They range between 4 and 20; high values reflect a more favorable mood state. In a healthy population, mean values are 15.0 (GM), 13.0 (AT), and 13.8 (CN) [30].

### 2.6. Data Analysis

SPSS version 17.0 was used for data analyses. For evaluation of safety and treatment effects intent-to-treat (ITT) analyses were used, which included all patients completing I1. Numbers of patients (*N*) in tables and figures are used for ITT analysis, respectively. Missing values of responses (≤10%) were substituted by estimated values procedure using the EM-algorithm of the program NORM Version 2.03 [31, 32] and the missing values procedure of SPSS version 17 [33]. All study variables were evaluated using standard descriptive statistics. Estimation of significant differences was performed by Student's *t*-test, Wilcoxon test, or ANOVA, as appropriate; for variables with high repetition, Friedman test was applied. *P* < 0.05 was considered significant in all tests. 

## 3. Results

**Figure 1:**
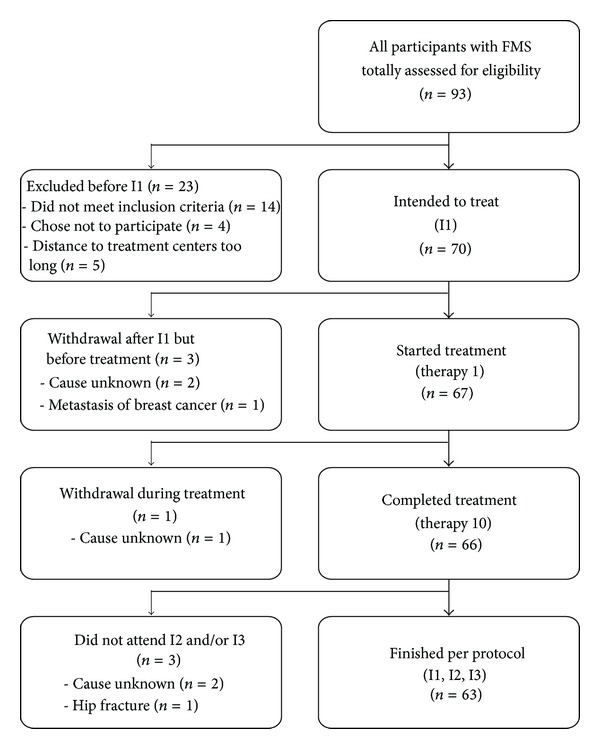
Flow diagram of the study. I1: before treatment; I2: 2–4 weeks after the last treatment session; I3: 2 months after I2.

Seventy patients were enrolled at I1; 66 completed all 10 treatment sessions. One patient dropped out after 5 treatment sessions due to unknown reasons, and 63 patients (90%) completed the study per protocol (Figure 1). The patients' characteristics at allocation (I1) are shown in Table 2. All were Caucasian; the age was 57.33 ± 10.48 years (mean ± SD). Primary FMS had been diagnosed 8.27 ± 6.11 years ago. The MPSS was calculated as 2.64 ± 0.48, indicating a high stage of chronicity.

Seventy patients were included in the safety analysis. Before the first treatment session 3 patients dropped out, one because of an acutely diagnosed metastasis of breast cancer and 2 did not show up for treatment for unknown reasons. At I2, 63 unwanted events were reported, which were clearly temporally related to treatment and/or were unfamiliar to the patients. Therefore, they were classified as adverse events, none being serious (Table 3). Twenty-nine patients (41.4%) did not report any unwanted events.

The physiotherapists rated the mean tolerability of the treatment (sessions 1–10) as 1.81 (95% confidence interval (CI) 1.77; 1.85) and the patients as 1.64 (1.59; 1.70) (*P* < 0.001). At I2, patients rated tolerability of the treatment as 1.8 (1.53; 2.07). At I2, 50 from 65 patients (76.9%) would have agreed to a further treatment series and at I3 49 from 63 patients (77.8%).

**Figure 2:**
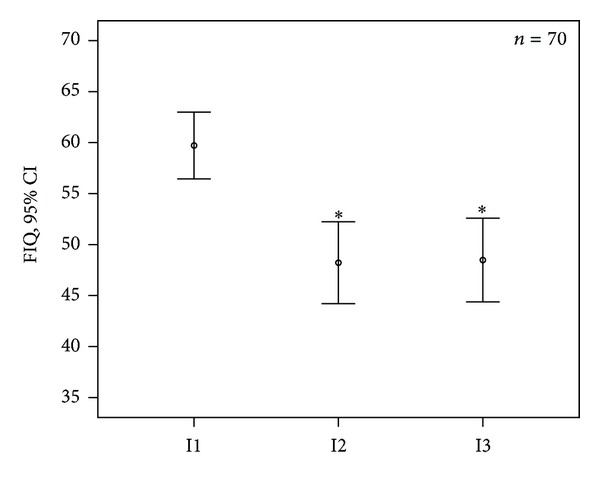
Fibromyalgia Impact Questionnaire (FIQ) before treatment (I1), 2–4 weeks after the last treatment session (I2), and 2 months after I2 (I3); mean and 95% confidence interval (95% CI); *P < 0.001: I1 versus I2 and versus I3 (*n* = 70).

The FIQ score was reduced at I2 by 19.3% and at I3 by 18.8% (*P* < 0.001 (Figure 2, Table 7)); between I1 and I2 or I3 also a shift from severe to moderate FIQ category occurred (Table 4). 

**Figure 3:**
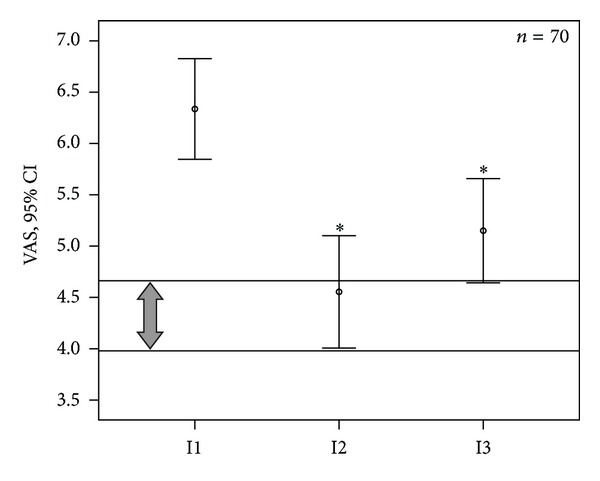
Pain (visual analogue scale (VAS)) before treatment (I1), 2–4 weeks after the last treatment session (I2), and 2 months after I2 (I3); mean and 95% confidence interval (95% CI); I1 versus I2 or versus I3: *P < 0.001; arrow and lines indicate 95% CI of the individually acceptable pain level of the patients.

Compared to I1, mean VAS score (pain) at I2 was reduced by 28.2% and at I3 by 18.8% (*P* < 0.001; Figure 3, Table 7). The patients' distribution according to the pain severity categories was mild (17.1%), moderate (25.7%), severe (35.7%), and very severe (21.5%) at I1; at I2 and I3, a shift to lower categories was shown (Table 5). At I1, the range of the individually acceptable VAS level for pain was 4.32 (95% CI: 3.98; 4.67) (arrow in Figure 3), which was below the range of the initial VAS level (*P* < 0.001). At I2, both levels did not differ, and 47.1% of the patients had reached their individually acceptable VAS level; at I3, this was true still for 41.4% (Figure 3). Compared to session 1, VAS was reduced at sessions 5 and 10 before and after treatment (Table 6). 

The scores of the subscales of the SF-36 of the patients were reduced at I1 compared to healthy, age-matched individuals (*P* < 0.01, data not shown). At I2 and I3, the scores of nearly all subscales were improved (Table 7). Compared to I1, affective and sensory scores of the SES were reduced at I2 and I3 (*P* < 0.001; Table 7). Also, two score values of the dimensions of the MBDF were significantly improved after treatment sessions 1 and 10 and after 10 sessions (*P* < 0.01) (Table 8).

At I1, 67 patients reported pharmacological treatment of FMS: analgesics (*N* = 27), antidepressants (*N* = 25), and other FMS-related drugs (*N* = 18). At I3, 61 patients (−9%) received pharmacological treatment and 6 patients had stopped taking analgesics (reduction: 22.2%). Forty-five patients used prescribed nonpharmacological treatment at I1: sports therapy (aqua jogging in warm water, 1/week, *N* = 30), myofascial release therapy (2/week, *N* = 11), transcutaneous electrical nerve stimulation (TENS, daily self-application, *N* = 7), and Swedish massage (2/week, *N* = 3). At I3, 26 patients (−44.6%) used prescribed nonpharmacological treatment: sports therapy (aqua jogging in warm water, 1/week, *N* = 19), myofascial release therapy (2/week, *N* = 14), TENS (daily self-application, *N* = 2), and Swedish massage (2/week, *N* = 5). Body acupuncture (2/week), which is not reimbursed by the health insurances, was used by 2 patients at I1 and by 3 patients at I3.

## 4. Discussion

The results suggest that deep oscillation massage is safe and well tolerable in patients with FMS. It even might be an efficacious single modality treatment, which should be confirmed in a controlled study. For comparison and discussion on external evidence of the results of the present study, clinical trials on pharmacological and nonpharmacological single-modality treatments were recommended in the German S3-guideline on FMS [2], and, additionally, clinical trials on manual therapy were searched for reports on safety, tolerability, and compliance. 

In a systematic review on amitriptyline, the mean adverse event rate of 25–50 mg daily amitriptyline was 51.8% and that of placebo 36.6%. The adverse events were mainly well-known drug-related side effects, and they were mild or moderate and mostly long-lived. Dropout rates were 25% (amitriptyline) and 30% (placebo) [34]. In a 6-month study, 6.1% of the patients on 25 mg amitriptyline/day and 19% of the patients using 2 × 10 min physical training plus aerobic session/day were lost to followup. Compliance to treatment on average was 75% of days in the amitriptyline group and 78% of days in the exercise group [35]. 

Studies on massage therapy in FMS reporting data on safety, tolerability, or compliance are rare. In a random study on connective tissue massages (15 sessions within 10 weeks), 28 of the 34 patients (83%) experienced the massage as being painful; however, 97% of all patients were willing to continue. One withdrew due to adverse events [36]. In a 3-week random study, the dropout rates were 8% (5 sessions of manual lymph drainage) and 4% (5 sessions of connective tissue massage). Adverse events were not recorded [37]. After 15 sessions of 35 min mechanical massage once per week, 9 out of 10 patients were willing to continue; adverse events were not recorded [38].

In the present study with 70 patients, 41.4% did not report any adverse events. The 63 mild and short-lasting adverse events were mostly worsening of prevalent symptoms in FMS [2]; one patient withdrew during treatment for unknown reasons. Self-reported tolerability immediately after each treatment session and at I2 was rated as good or very good, and most patients were willing to continue the therapy. Altogether, safety and tolerability of deep oscillation massage seem to be superior to amitriptyline or connective tissue massage [36, 38]. 

The secondary outcome parameters of the present study were chosen in order to allow preliminary comparisons with controlled studies (FIQ, pain (VAS), SF-36), to address the patient-centered approach (individually acceptable pain level), and to gain information on a possible influence of the therapy with deep oscillation massage on pain perception and mood aspects (SES, MDBF). 

The FIQ is widely used in clinical studies on FMS [39]. In a 6-month study, 25 mg/day amitriptyline or 2 × 10 min physical training plus aerobic sessions/day improved the FIQ score by 22.9% and 23.2%, respectively [35]. In a 12-week study comparing aquatic exercises (3/week) in warm seawater versus in a warm swimming pool, all patients additionally performed a 60 min physical fitness program. In the seawater group, the FIQ score was reduced by 47.8% and in the pool group by 45.8% [40]. In a 3-week randomized study, manual lymph drainage or connective tissue massage (5/week) reduced the FIQ score by 60.5% or 42.3% [37]. In a further study on connective tissue massage, 15 sessions within 10 weeks reduced the FIQ score by 17.1% and in the waiting list group by 4.1% [36]. In a randomized study, patients performed either 3 × 60 min low-impact exercises per week or received a diet recall once a week for 6 months. The FIQ was reduced by 10.7% in the exercise group and by 9.2% in the diet group [41]. A reduction by 19.3% at the posttreatment and by 18.8% at the follow-up visit seems to indicate treatment effects being superior to placebo and low-impact exercise and in the range of pharmacologic interventions, rehabilitation programs, or massage-related techniques.

In a meta-analysis of 21 clinical trials on FMS pain response was analyzed as an improvement of at least 30% from baseline [42]. In a 12-week study on amitriptyline (25–37.5 mg/day), VAS was reduced by 25.0% and in the placebo group by 8.8% [43]. In patients performing 3 × 60 min low-impact exercises/week for 6 months, VAS was reduced by 20.0% and in the control group (weekly diet recall) by 9.0% [41]. In the study by de Andrade et al., VAS was reduced by 40.7% in the sea-water group and by 38.3% in the pool group [40]. In a 3-week study on 15 sessions of 45 min manual lymph drainage therapy or 10 min connective tissue massage, VAS was improved by 88.6% and by 60.3%, respectively [37]. In a 10-week study, after 15 sessions of connective tissue massage, VAS was reduced by 34.6% and at the 3-month followup by 18.8% [36]. Twelve sessions of 60 min lymph drainage (3/week) reduced VAS by 32.5% and at the 5-month followup by 18.7% [44]. In the present study, the improvement of VAS (28.2% at I2, 18.8% at followup) was in the same range as most interventional studies and comparably sustained. It should be mentioned that in all studies cited above both FIQ and VAS were measured immediately after the last intervention, while in the present study they were rated about two weeks later. Presumably the improvement might be underestimated. Also duration and stage of chronicity of FMS differed in the above-mentioned studies, thereby allowing only a rough comparison to the results of our study. Also, the various forms of massage applied may have contributed to the differing study results. 

Patients with FMS show reduced health status domains of the SF 36 compared to the general population [45, 46]. In a pooled analysis of 4 studies with duloxetine (120 mg/daily; 12 or 28 weeks), the pcs of the SF 36 was improved by 14.3% and in the placebo group by 10.6%; the mcs was improved by 10.3% and by 3.7%, respectively [47]. In the study by de Andrade et al., the pcs of the sea-water group was improved by 46.5% and the mcs by 45.6% and in the pool-water group by 37.1% (pcs) and 37.5% (mcs) [40]. In a two-month study with weekly sessions addressing health promotion by life-style adjustment, exercise and physical activity, stress management, and improvement of personal relationships, the pcs was improved in the 3-month follow-up visit by 5.6% and the mcs by 4.0% [48]. In the present study, the initial SF 36 score values were in the published range of FMS patients; it was improved comparably to treatment with duloxetine but was superior to placebo [47].

The effects of deep oscillation massage on pain perception and mood were tested with the SES and the MDBF. In a controlled trial the affective part of the SES was reduced by 16.7% after an inpatient rehabilitation program and by 8.4% at the three-month follow-up and the sensory part by 6.4% and −1.8% [49]. In the present study, the effects on the SES scores were comparable, however, more sustained. Also, all dimensions of MDBF which were initially below those of a healthy population were rapidly improved and reached normal levels [30, 50]. Since negative affectively charged experiences seem to amplify pain processing in the central nervous system, contributing to the development and maintenance of fibromyalgia symptoms, further investigations on treatments which seem to be able to modify pain perception and mood may be useful [51]. 

In an observational study, concomitant therapies cannot be controlled. They, therefore, may have influenced the results. The pharmacological therapy, especially analgesics, was reduced by 22.2% at followup compared to the initial visit. This presumably is a consequence of the pain reduction. 

The changes in concomitant prescribed physical therapies and acupuncture during the study course need a detailed elucidation. In Germany, refundable physical therapies are lowly budgeted generally. Therefore, patients have difficulties to get a prescription, especially a prolongation. This presumably is the background why 44.8% of the patients of this study used aqua jogging at the initial visit (I1) but only 31.2% at followup (I3). This, however, does not imply that they did not perform any aerobic endurance training on their own during the study, when they felt better and had less pain. The high MPSS of our patients demonstrates that they had obtained at least one inpatient rehabilitation. In Germany, one of the standard rehabilitation modules is “education to cope with FMS.” This comprises the elucidation of the disease model which includes that they suffer from a pain condition in consequence of long-term dysfunctional stress management. The patients of our study, therefore, knew that self-management of stress and self-performed aerobic exercises are the first-line treatment and necessary for further amelioration.

The changes in the concomitant passive nonpharmacological therapy presumably did not influence the results of the present study. There is still no convincing study available indicating that myofascial release therapy could improve FIQ or pain in FMS patients. The percentage of patients, who received myofascial release therapy (16.4%) at I1, was increased by 6.5% at I3. Also Swedish massage and TENS presumably have nearly no influence on the course of FMS in the present study. Swedish massage increased from 4.5% of the patients to 8.1%. The percentage of TENS was even reduced from 10.5% to 3.0%.

Body acupuncture with the indication “FMS” is not reimbursed by German health insurances. This is reflected by the initially very low treatment rate (3.0%), which was increased marginally to 4.9% at the final visit. 

The present study has several methodological weaknesses and limitations. Obviously, results of randomized trials and observational studies cannot be compared directly. Due to the lack of a control group, a nonspecific and placebo effects of deep oscillation massage on symptoms and quality of life can bias the results. However, the analysis of the effects of placebo on various scores in the controlled trials cited revealed that they were much lower than the treatment effects shown in the present study. 

Rare adverse events of deep oscillation massage were not detected due to the small number of patients included. Therapists' attention, expectancy, and time effects were not controlled. This can result in the undervaluation of reported symptoms. Furthermore, a sampling bias leading to an easier-to-treat sample population could be discussed. However, the patients of the present study had a high stage of chronicity (MPSS) and thereby should be much less responsive to any therapy than those with a low stage of chronicity [19, 20]. An unspecific spontaneous improvement sustaining for at least 9 months cannot entirely be excluded, although longitudinal studies indicate an absence of spontaneous improvement in symptoms or remission in the natural course of FMS [52]. 

Due to the control visit (I2) 2–4 weeks after the final treatment session, we may have missed the maximum effect on the endpoints. On the other hand, this time schedule allowed a preliminary estimation of the long-term effect, since the follow-up visit took place 2 months after the control visit. 

Deep oscillation massage might have been a diversion from self-management of stress in these patients who suffered from FMS since several years which might explain the high rate of unemployment or retirement. This could be addressed in a controlled study. It is also unknown whether the subjective effects of deep oscillation massage differ from those of Swedish massage or myofascial release therapy. 

The massage device is designed preferably for self-application. In the present study, it was applied by trained physiotherapists in order to reduce the variance of treatment. Also, self-application on the back and neck seems to be difficult and thereby may not allow sufficient relaxation. The comparison of the effects of self-application and treatment by a skilled person including a cost analysis should be investigated in a further study.

## 5. Conclusions

In this pilot study, deep oscillation massage was shown to be safe and well tolerated in patients with moderate to severe grades of FMS of high chronicity and was highly accepted. The 3-month follow-up revealed sustained improvements in symptoms and quality of life, hence suggesting a benefit from treatment with deep oscillation massage, even though it was applied as a single-modality treatment. It also should be assessed whether vibration massage is a useful completion of aerobic exercise. This should be confirmed by a controlled clinical trial. 

## Figures and Tables

**Table 1:** Secondary outcome measures of the study before treatment (I1), 2–4 weeks after the last treatment session (I2), and 2 months after I2 (I3).

Outcome measures	I1	Before/after treatment										I2	I3
		1	2	3	4	5	6	7	8	9	10		
FIQ and checkup	X											X	X
VAS	X	X	X	X	X	X	X	X	X	X	X	X	X
SF 36	X											X	X
SES	X											X	X
MDBF		X									X		

FIQ: fibromyalgia impact questionnaire, VAS: pain (visual analogue scale), SF 36: quality of life, SES: pain perception scale, MDBF: multidimensional mood state questionnaire.

**Table 2:** Characteristics at allocation.

Characteristics	*N* = 70	%
Female	68	97.1
Married/partnered	51	72.9
Employment status		
Employed	19	27.1
Retired	45	64.3
Unemployed	6	8.6
Education		
University degree		
Yes	22	31.4
No	38	54.2
Unknown	10	14.2

**Table 3:** Adverse events reported at I2 (2–4 weeks after the last treatment session).

Adverse events reported at I2	# of events	% of events
Pain during or immediately after therapy	18	28.6
Fatigue immediately after therapy	13	20.6
Stranguria/rectal tenesmus immediately after therapy	6	9.5
Nausea immediately after therapy	5	7.9
Adynamia immediately after therapy	3	4.8
General tension immediately after therapy	2	3.2
Pruritus immediately after therapy	2	3.2
Metastasis of breast cancer: (w)	1	1.6
Once-only reported events*	13	20.6

Total	63	100.0

(w): withdrawal before treatment; #: number.

*Flatulence, swollen hands, muscle cramp, irritated skin, weight reduction, broncholysis, vomiting, vertigo, increased production of bronchial mucus, dry eyes, asthmatic attack, recrudescence of rosacea, and sleep disturbance.

**Table 4:** Distribution of patients (*N* = 70) according to the Fibromyalgia Impact Questionnaire (FIQ) severity categories before treatment (I1), 2–4 weeks after the last treatment session (I2), and 2 months after I2 (I3).

FIQ	I1	I1 (%)	I2	I2 (%)	I3	I3 (%)
0 to <39	6	8.6	9	12.9	12	17.1
39 to <59	25	35.7	40	57.1	38	54.3
59 to 100	39	55.7	21	30.0	20	28.6

**Table 5:** Distribution of patients (*N* = 70) according to the pain (visual analogue scale (VAS)) severity categories before treatment (I1), 2–4 weeks after the last treatment session (I2), and 2 months after I2 (I3).

VAS	I1	I1 (%)	I2	I2 (%)	I3	I3 (%)
0.0 to 3.9	12	17.1	27	38.6	21	30.0
4.0 to 5.9	18	25.7	22	31.4	27	38.6
6.0 to 7.9	25	35.7	16	22.9	16	22.9
8.0 to 10.0	15	21.5	5	7.1	6	8.5

**Table 6:** Before and after treatment sessions 1, 5, and 10: pain (visual analogue scale (VAS)); mean and 95% confidence interval (95% CI).

Outcome measure *N* = 67	Treatment sessions		
	1	5	10
VAS			
Before treatment	6.33 (5.86; 6.80)	5.24 (4.84; 5.63)^~^	4.74 (4.22; 5.25)^~^
After treatment	5.15 (4.65; 5.65)^+^	4.30 (3.86; 4.74)^+∗^	3.83 (3.32;4.34)^+#^

^+^Pre/posttreatment: *P* < 0.001.

^~^Session 1/session 5 before treatment and session 1/session 10 before treatment: *P* < 0.001.

*Session 1/session 5 post treatment: *P* < 0.01; ^#^session 1/session 10 post treatment: *P* < 0.001.

**Table 7:** Fibromyalgia Impact Questionnaire (FIQ), pain (visual analogue scale (VAS)), quality of life subscales of SF-36 (physical functioning index (pfi), role-physical index (rolph), body-pain index (pain), general health perceptions index (ghp), vitality index (vital), social functioning index (social), role-emotional index (rolem), mental health index (mhi), health transition item (rawhtran), physical component summary (pcs) and mental component summary (mcs)), and pain (SES; affective and sensory scores (*T*-values)) before therapy (I1), after therapy (I2), and 2 months after I2 (I3) with deep oscillation massage; mean and 95% confidence interval (95% CI).

Outcome measures *N* = 70	I1	I2	I3	*P* value
FIQ	59.7 (56.5; 63.0)	48.2 (44.2; 52.2)	48.5 (44.4; 52.6)	<0.001^#^

VAS	6.34 (5.85; 6.83)	4.55 (4.01; 5.10)	5.15 (4.64; 5.66)	<0.001^#^

SF 36				
pfi	45.7 (40.5; 50.9)	54.8 (49.0; 60.4)	52.1 (46.7; 57.5)	<0.002^#^
rolph	16.1 (9.6; 22.5)	35.0 (26.1; 43.9)	30.0 (21.2; 38.8)	<0.005^#^
pain	32.1 (28.7; 35.4)	42.4 (38.4; 46.4)	41.7 (37.0; 46.3)	<0.001^#^
ghp	39.4 (35.9; 42.9)	45.8 (42.0; 49.6)	43.7 (39.8; 47.6)	<0.009^#^
vital	32.9 (28.9; 36.9)	41.2 (36.7; 45.7)	40.4 (35.7; 45.0)	<0.001^#^
social	54.3 (47.7; 60.8)	62.0 (56.1; 67.9)	60.4 (54.5; 66.2)	<0.009*
rolem	42.9 (32.0; 53.7)	52.4 (41.8; 63.0)	43.8 (33.6; 54.0)	n. s.
mhi	52.2 (47.9; 56.5)	61.8 (58.0; 65.6)	56.3 (51.9; 60.7)	<0.05^#^
rawhtran	3.31 (3.05; 3.58)	2.56 (2.31; 2.80)	2.71 (2.45; 2.97)	<0.001^#^
pcs	30.9 (29.1; 32.7)	35.0 (33.0; 37.0)	34.8 (32.7; 37.0)	<0.001^#^
mcs	40.5 (37.5; 43.4)	44.1 (41.5; 46.7)	41.7 (38.9; 44.4)	<0.02*

SES				
Affective score	52.3 (50.0; 54.7)	45.5 (43.2; 47.7)	47.0 (44.4; 49.7)	<0.001^#^
Sensory score	55.8 (53.2; 58.4)	51.0 (48.3; 53.7)	50.3 (47.8; 52.8)	<0.001^#^

All of the data shown are mean (95% CI).

^#^Significant difference between I1 and I2 and between I1 and I3 (the weaker *P* value is given).

*Significant difference between I1 and I2 only; n. s.: not significant (*P* > 0.05).

**Table 8:** Multidimensional Mood State Questionnaire (MBDF), short-form versions A and B for pre- and post-treatment values, respectively; mean and 95% confidence interval (95% CI) of the dimensions good/bad mood (GM), feeling awake/tired (AT), and calm/nervous (CN).

*N* = 67	Treatment session 1			Treatment session 10		
	GM	AT	CN	GM	AT	CN
Pre (A)	11.7 (10.8; 12.6)	10.2 (9.3; 11.1)	11.6 (10.8; 12.4)	13.8* (12.9; 14.8)	12.5** (11.6; 13.4)	13.5** (12.7; 14.2)
Post (B)	14.7^+^ (14.0; 15.5)	12.6^+^ (11.6; 13.5)	14.7^+^ (13.8; 15.5)	16.2^∗+^ (15.3; 17.0)	13.0 n. s. (11.9; 14.2)	16.3^∗∗+^ (15.5; 17.0)

Pre-/posttreatment: ^+^ *P* < 0.01; n. s.: not significant (*P* > 0.05).

Session 1 before/session 10 before or session 1 post treatment/session 10 post treatment: *P < 0.01, **P < 0.001.
